# Pulmonary Artery Sling Blurs the Distal Trachea on Frontal Chest Radiography

**DOI:** 10.5334/jbsr.3040

**Published:** 2023-03-06

**Authors:** Quentin Jacquemin, Alain Nchimi

**Affiliations:** 1 Centre Hospitalier de Luxembourg, LU

**Keywords:** Lung, pediatrics, bronchus, trachea, artery sling

## Abstract

**Teaching Point:** The pulmonary artery sling can be suspected on frontal chest radiography, not only by ancillary findings like lobe/lung emphysema or persisting atelectasis, but also blurring of the distal trachea or carina.

## Case History

**Figure 1:**
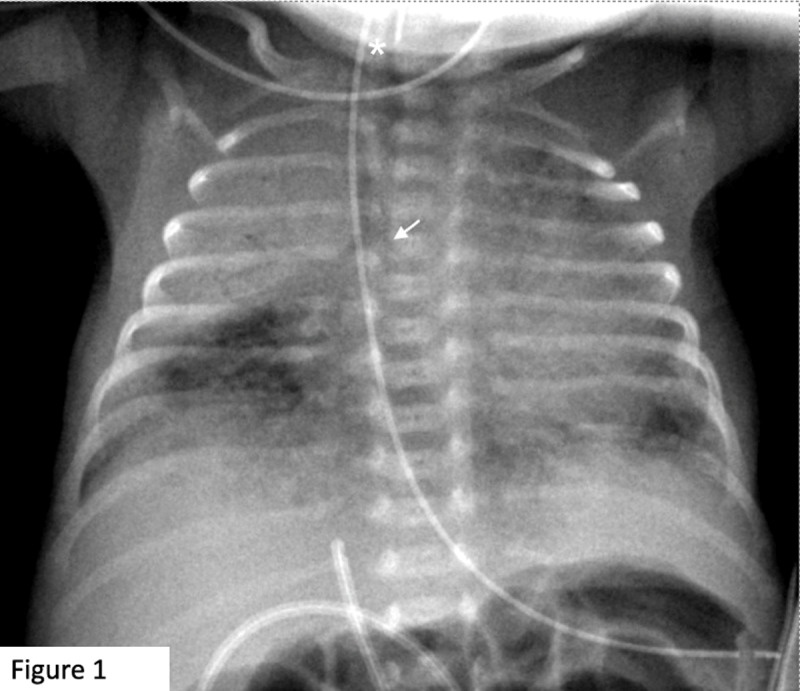


We report the case of a premature newborn, 30 weeks of gestation, without any noticeable birth event. On day one, he experienced respiratory stridor, polypnea, and intercostal retractions. Anteroposterior chest radiograph (CRX) showed right upper lobe atelectasis with air bronchogram. As his condition did not improve, an endotracheal tube was placed on day two. On control CRX (Figure 1), the tube was in an abnormally high position (asterisk), approximately 10mm under the vocal cords. The trachea was displaced to the right due to the right upper lobe atelectasis. Both the carina and the left main bronchus were not clearly depicted (arrow).

**Figure 2:**
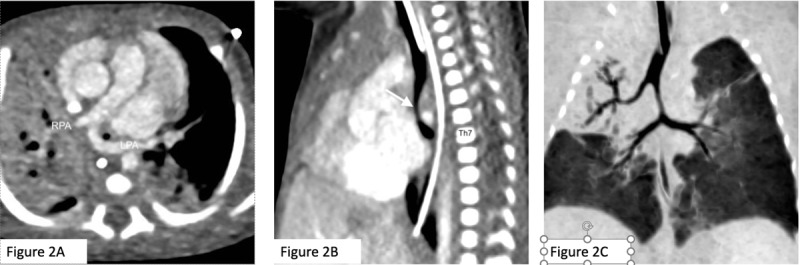


Following axial computed tomography (CT) angiography (Figure 2A), an aberrant origin of the left pulmonary artery (LPA) from the right pulmonary artery (RPA) was seen, with a subsequent posterior compression of the trachea on sagittal reformation (Figure 2B, arrow), causing a tight stenosis of the pre-carinal trachea. The carina is located at the level of the seventh thoracic vertebral body. Coronal reformation (Figure 2C) shows an abnormal origin of the right upper lobe bronchus on the right, with abnormal narrowing of the tracheal segment below this origin, due to the abnormal origin and course of the LPA.

## Comments

The term LPA ‘sling’ comes from the fact that it originates from the RPA and crosses the sagittal midline in between the trachea and esophagus to reach the left lung hilum. This causes a tracheal stenosis. A posterior mass effect on the lateral CRX is the most widely acknowledged finding related to this vascular anomaly. Nevertheless, lateral CRX is being progressively abandoned in neonates because of technical difficulties and radiation burden.

Common but nonspecific findings for LPA sling on anteroposterior CRX include a lobar emphysema/atelectasis (most often the right upper lobe) persisting over time despite therapeutic measures. Herein we reported that undepictable trachea could be added in combination to these findings, to help raise the suspicion index for LPA sling for two reasons. First, the double superimposition of the pulmonary vasculature on the anteroposterior projection causes increased opacity and blurring of the right stripe of the distal trachea or the carina (depending on the level of the sling). Second, congenital bronchial malformations, potentially associated with pulmonary artery sling, such as displaced carina in this case, also contribute to a failure of depiction on anteroposterior CRX. Though valuable in terms of specificity for a mediastinal vascular ring, the identification of a blurred distal trachea (or carina) may lack sensitivity, given the common presence of nasogastric and/or endotracheal tubes and the displacement of the trachea over the lateral vertebral elements.

According to the Wells and Landing’s classification, there are two types of LPA sling depending on the position of the artery in relation to the carina. In Type 1, the carina is at its usual level (i.e., the fourth and fifth thoracic vertebral bodies), and the tracheobronchial anatomy is normal. In Type 2, the carina is located lower, at the level of the sixth and seventh thoracic vertebral bodies, the angle of the bronchial bifurcation is widened, and the bronchial anatomy can be altered, as in our patient, who had a type 2A malformation, as he additionally had a right tracheal bronchus [1].
